# On Artificial Soda Water

**Published:** 1815-10

**Authors:** Robert Bakewell

**Affiliations:** of Tavistock-Street, London.


*For the London Medical and Physical Journal.*

### *On Artificial Soda Water;* by Robert Bakewell, Esq. of Tavistock-street, London.

MUCH of the soda water now sold is made in copper vessels; and the carbonic acid, in its concentrated state, is a powerful solvent of that metal. Another improvement in the preparation of soda water consists in passing the carbonic acid through an alkaline solution, in a separate vessel sel, by which any sulphuric acid, or sulphureous acid vapour, which might pass over in the common method of preparing soda water, is intercepted, and the gas rendered perfectly pure.

The alkalescent soda water contains a larger portion of supercarbonate of soda, with a few grains of supercarbonate of ammonia, which the briskness of the water renders nearly insensible to the taste.

The saline aperient waters contain sulphate of magnesia and soda, and a small quantity of carbonate of magnesia held in solution, from one to three drams of salt in each bottle.

